# Development of a first-in-class antibody and a specific assay for α-1,6-fucosylated prostate-specific antigen

**DOI:** 10.1038/s41598-024-67545-1

**Published:** 2024-07-17

**Authors:** Steinar Halldórsson, Lars Hillringhaus, Caroline Hojer, Andrea Muranyi, Michael Schraeml, Magdalena Swiatek-de Lange, Gloria Tabarés

**Affiliations:** 1 Pharma Research and Early Development, F. Hoffmann-La Roche AG, Basel, Switzerland; 2 Roche Diagnostics GmbH, Nonnenwald, Penzberg, Germany; 3 Roche Diagnostics Solutions, Tucson, AZ USA

**Keywords:** Diagnostics, Biomarkers

## Abstract

Prostate-specific antigen (PSA) levels are widely used to screen for prostate cancer, yet the test has poor sensitivity, specificity and predictive value, which leads to overdiagnosis and overtreatment. Alterations in the glycosylation status of PSA, including fucosylation, may offer scope for an improved biomarker. We sought to generate a monoclonal antibody (mAb) targeting α-1,6-fucosylated PSA (fuc-PSA) and to develop a tissue-based immunological assay for fuc-PSA detection. Immunogens representing fuc-PSA were used for immunisation and resultant mAbs were extensively characterised. The mAbs reacted specifically with fuc-PSA-specific glycopeptide, but not with aglycosylated PSA or glycan without the PSA peptide. Reactivity was confirmed using high-throughput surface plasmon resonance spectroscopy. X-ray crystallography investigations showed that the mAbs bound to an α-helical form of the peptide, whereas the native PSA epitope is linear. Protein unfolding was required for detection of fuc-PSA in patient samples. Peptide inhibition of fuc-PSA mAbs was observed with positive screening reagents, and target epitope specificity was observed in formalin-fixed, paraffin-embedded tissue samples. This research introduces a well-characterised, first-in-class antibody targeting fuc-PSA and presents the first crystal structure of an antibody demonstrating glycosylation-specific binding to a peptide.

## Introduction

Early detection of prostate cancer currently relies on abnormal digital rectal examination or elevated levels (10 ng/mL or higher) of prostate-specific antigen (PSA)^1,2^. While prostate cancer screening with PSA has led to a reduction in advanced disease and disease-specific mortality, the PSA test shows poor sensitivity and specificity^3–6^. PSA is a serine protease member of the human kallikrein family that circulates in blood as free PSA or complexed to α1-antichymotrypsin and α2-macroglobulin^1,7–9^. PSA is not a cancer-specific biomarker as it is produced in both normal and cancerous prostate tissue and secreted into seminal fluid; it is therefore challenging to discriminate between prostate cancer and other prostatic diseases (e.g., benign prostatic hyperplasia and prostatitis) using PSA alone^1,7,8^. This is particularly evident in what is termed the “grey area”, corresponding to a blood PSA concentration of 4–10 ng/mL^9^.

These PSA test limitations can lead to overdiagnosis of prostate cancer, whereby cancers are being detected that would otherwise not manifest clinically over a patient’s lifetime^3^. This, in turn, can lead to unnecessary biopsies and overtreatment of men with a low risk of disease progression^3^. These men will incur the side effects that are associated with prostate cancer therapy (e.g., urinary incontinence and erectile dysfunction), despite not needing the treatment^3,5,10^. There is, therefore, an unmet need for improved prostate cancer biomarkers that can differentiate aggressive cases of prostate cancer. It has been suggested that the glycosylation status of PSA might present a promising marker for recognition of aggressive prostate cancer^6,10–13^.

Glycosylation is a common post-translational modification that is functionally important for proteins involved in several physiological processes^13^. Altered glycosylation patterns, including altered fucosylation and sialylation, have been reported in cancer cells compared with healthy cells^13–16^. Fucosylated glycans can be characterised as core type (α-1,6-), H-type (α-1,2-) or Lewis type (α-1,3-/α-1,4-) fucoses^6^. Increased core fucosylation is observed in serum during the process of carcinogenesis and is recognised in various types of cancer, such as hepatocellular carcinoma (HCC), pancreatic cancer and prostate cancer^9,17,18^.

Human PSA has a single *N*-glycosylation site at asparagine (Asn) 69 comprising a core α-1,6-fucose^6,9^. Studies using lectin-based and mass spectrometry (MS) approaches have assessed altered glycosylation of PSA in the sera of cancer patients^4–6,19,20^. Lectin-based assay systems are relatively straightforward: glycans are detected using lectins that recognise carbohydrate moieties^4,21–23^. However, lectin-based approaches do not recognise protein moieties and generally exhibit poor sensitivity and specificity^1,9,24^. MS and liquid chromatography approaches may also be employed for analysis and quantification of serum and seminal plasma glycosylated PSA, but these approaches have either failed to show improved diagnostic power compared with conventional PSA measurements^1^, or are not feasible in the clinical setting due to their complexity and prolonged measuring times^6^.

An alternative approach is antibody-based assays; however, generation of high-affinity antibodies to carbohydrates is challenging because of their low immunogenicity^17,24^. This is likely due to known issues with the immunogenicity of glycan structures (e.g., α-1,6-fucose), which are often highly conserved in animals used for immunisation^17^. Moreover, antibodies against carbohydrates often have very low affinities and may lack sufficient specificity^24^. Monoclonal antibodies (mAbs) generated against α-2,3-linked sialic acid—a terminal *N*-glycan structure that has been identified on the PSA of patients with prostate cancer—have been used to quantify PSA^10^. To date, there are no reports of mAbs that specifically recognise both carbohydrate and peptide components of PSA. However, antibodies recognising fucosylated α-fetoprotein (AFP), a well-known HCC biomarker, have been described^17^.

The aim of the current manuscript is to describe and characterise a first-in-class antibody directed to α-1,6-fucosylated PSA (fuc-PSA), which is novel in recognising both carbohydrate and peptide components of PSA; we also describe the development of a specific assay for the detection of fuc-PSA.

## Methods

### Ethics

The study was conducted in accordance with the Declaration of Helsinki, and the protocol was approved by the Ethics Committee of Ethikkommission der Landesärztekammer Rheinland-Pfalz and Charité—Universitätsmedizin Berlin Ethikkommission (Project identification code: NT-MIP-PC-01). All experiments were performed in accordance with the ARRIVE guidelines (Animal Research: Reporting of In Vivo Experiments).

### Immunogen preparation

Peptides and glycosyl azides were synthesised according to published methodology^25–27^. The following were prepared: PSA(67–79)-G0F, a glycopeptide consisting of amino acids 67–79 of PSA containing the Asn-69 *N*-glycosylation with an octasaccharide with a core α-1,6-fucose (G0F); PSA(67–79)-G2, a glycopeptide with the same peptide sequence containing an Asn-69 glycan with a nonasaccharide and lacking the core α-1,6-fucose (G2); and Asn-G0F, an octasaccharide with a core α-1,6-fucose (representing glycan without the PSA peptide [aa 67–79]) linked to an asparagine residue. Standard building blocks for solid-phase peptide synthesis were purchased from Merck; disaccharides for glycan synthesis were purchased from Sussex Research Laboratories Inc.

Peptides with mono- and disaccharide (GlcNAc and Fuc-GlcNAc) were synthesised by solid-phase peptide synthesis. Fluorenylmethoxycarbonyl (Fmoc) -protected glycoamino acids (1.2 equivalents) were coupled for 1 h in dimethylformamide with 1.2 equivalents hexafluorophosphate azabenzotriazole tetramethyl uronium (HATU), 1.2 equivalents hydroxy azabenzotriazole (HOAt) and 10 equivalents of N,N-diisopropylethylamine (DIPEA) relative to resin loading. After, trifluoroacetic acid (TFA)-cleaved peptides were dissolved in methanol, and sodium methanolate was added dropwise until pH 10 was reached. The solution was stirred for 4 h and subsequently neutralised with acetic acid. After removal of the solvent, the peptides were purified by preparative RP-HPLC using a gradient of acetonitrile/water containing 0.1% TFA. The molecular weight of the peptides was confirmed by electrospray ionisation MS. Immunogens were prepared by coupling PSA(67–79)-G0F to keyhole limpet hemocyanin (KLH) as a carrier protein, as previously described^28^. Biotinylated peptides were prepared by attaching a glutamic acid derivative with a polyethylene glycol (PEG3)-biotin side chain to the N terminus during solid-phase peptide synthesis. The HPLC purities were as follows: KLH-G0F: 73%, KLH-G2: 70%, Biotin-G0F: 99%, Biotin-G2: 99%, Biotin-Fuc-GlcNAc: 90% and Biotin-PSA peptide without glycosylation: 77%.

### Immunisations

Three female New Zealand white rabbits, 12–16 weeks old, were immunised with KLH-PSA(67–79)-G0F. The animals were immunised weekly for the first month, and monthly thereafter. For the first immunisation, 500 µg of KLH-coupled peptide was dissolved in 0.9% NaCl and emulsified in 2 mL of complete Freund’s adjuvant. For all subsequent immunisations, complete Freund’s adjuvant was replaced by 1 mL of incomplete Freund’s adjuvant emulsion.

### Titre analysis

Titre analysis was performed according to a standard enzyme-linked immunosorbent assay (ELISA) protocol^29^. Serum titrations were performed using biotinylated PSA(67–79)-G0F as a positive control, and biotinylated PSA(67–79)-G2 as a negative control.

Biotinylated screening peptides were immobilised on the surface of 96-well streptavidin-coated microtitre plates by incubating 100 μL per well of a 16 ng/mL solution for 60 min at room temperature. Subsequent washing was performed using an automated instrument (Biotek) according to manufacturer’s instructions. A small amount of serum from each rabbit (2–3 mL per animal) was collected on day 35 and day 165 after the start of the immunisation campaign. The serum from each rabbit was diluted 1:300, 1:900, 1:2,700, 1:8,100, 1:24,300, 1:72,900, 1:218,700 and 1:656,100 with phosphate-buffered saline (PBS) containing 1.0% BSA. 100 μL of each dilution was added to the plate previously prepared with the screening peptides and incubated for 60 min at room temperature.

Bound antibody was detected with horseradish peroxidase (HRP)-labelled F(ab′)_2_ goat anti-rabbit Fcγ (Jackson ImmunoResearch Laboratories Inc) and ABTS (2,2ʹ-azinobis [3-ethylbenzothiazoline-6-sulfonic acid]-diammonium salt) substrate solution (Roche). The titre of the analysed animals was set at 50% signal decrease of the dilution curve.

### B-cell cloning

For enrichment of antigen-reactive B cells, 100 ng/mL biotinylated PSA(67–79)-G0F was pre-incubated for 15 min at 4 °C with 50 M peripheral blood mononuclear cells isolated from the peripheral blood of each immunised animal (incubation buffer PBS with 0.1% BSA)^30^. After a washing step, the peripheral blood mononuclear cells were incubated with streptavidin-coated beads (Miltenyi) in PBS with 2 mM EDTA for 15 min at 4 °C. Enrichment of antigen-positive B cells was performed using magnetic-activated cell sorting (MACS) columns (Miltenyi). B-cell single-cell sorting and culture was done as described previously but using MACS-enriched B cells^29^.

### ELISA

ELISA was used to identify B cells expressing antibodies with desired binding characteristics (i.e., binding to PSA[67–79]-G0F; not binding to PSA[67–79]-G2 and Asn-G0F). Biotinylated screening agents PSA(67–79)-G0F, PSA(67–79)-G2 and Asn-G0F were immobilised on the surface of streptavidin-coated 96-well plates (Nunc) by incubation of 100 µL PBS with 1.0% (w/v) BSA per well of 100 ng/mL solutions for 60 min at room temperature. The plates were washed and 30 µL of rabbit B-cell culture supernatant was transferred to each well and incubated for 1 h at room temperature. For the detection of antibodies bound to the screening agents, HRP-labelled goat anti-rabbit IgG (Jackson ImmunoResearch Laboratories Inc) for 60 min at room temperature in PBS with 1.0% (w/v) BSA. The plates were washed and then the ABTS substrate solution (Roche) was used according to the manufacturers’ instructions (20 min at room temperature)^31,32^.

### Molecular cloning and recombinant expression

Clones that bound to PSA(67–79)-G0F and discriminated from the negative screening agents according to the selected cut-offs (optical density [OD] > 0.6 in positive screening and < 0.6 in negative screening) were selected for subsequent molecular cloning and recombinant expression as previously described^29^. ELISA was repeated for those clones that were successfully cloned and for which the sequences encoding the variable-domain heavy (V_H_) and light chains (V_L_) could be unambiguously determined. This ELISA was performed using the supernatant of the recombinant expression from these clones and the more stringent cut-off criteria (OD > 1 for positive screening agent and OD at or below average background signal for negative screening agents).

### Kinetic analysis

Recombinant mAbs from the clones of interest were further investigated for their kinetic rate properties and antigen-binding specificity for fucosylated and non-fucosylated PSA-derived peptides. Kinetic analysis of the antibodies assisted by surface plasmon resonance spectroscopy was performed at 37 °C using a Biacore 8 k instrument (GE Healthcare) with a series S CM5 research-grade sensor, normalised in HBS-ET buffer (10 mM HEPES [4-(2-hydroxyethyl)-1-piperazineethanesulfonic acid] pH 7.4, 150 mM NaCl, 3 mM EDTA [ethylenediaminetetraacetic acid], 0.005% w/v Tween 20). The sample dilution buffer was also used as the system buffer (i.e., HBS-ET buffer supplemented with 1 mg/mL carboxymethyl dextran [SIGMA]).

Up to 10,000 RU polyclonal goat anti-rabbit immunoglobulin G (IgG; GARb-Fcγ, Jackson Laboratories) were immobilised on the sensor surface using EDC/NHS (1-ethyl-3-[3-dimethylaminopropyl] carbodiimide/N-hydroxysuccinimide) chemistry. Rabbit IgG antibody (150 kDa)-containing rabbit B-cell primary cell culture supernatants were diluted threefold with sample buffer and captured for 2 min under a flow rate of 5 µL/min. The antibody capture level was quantified in RU for each antibody. The non-fucosylated peptide, PSA(67–79)-G2 (3.7 kDa), and the fucosylated peptide, PSA(67–79)-G0F (3.9 kDa), were diluted in sample buffer to a concentration series of 0 (buffer), 11, 33, 100, 300 and 900 nM. The 100 nM samples were injected twice as control. The analytes were injected at 30 µL/min for 3 min association time. After the association phase of the 900 nM analyte injection, a report point (binding late) was set to quantify the signal response at antibody ligand saturation. Antibody–antigen dissociation was monitored for 5 min. The GARb-Fcγ antibody capture system was regenerated by injection of 10 mM glycine pH 2 at 30 µL/min for 1 min, followed by two consecutive 1 min injections of 10 mM glycine pH 2.25 at 30 µL/min.

Overlay plots of concentration-dependent antigen binding were generated, and kinetic rates were determined with the Biacore 8 k evaluation software (Cytiva, Biacore TM insight Control SW V3.0.11.15423 and Biacore TM insight Evaluation SW Version V3.0.11.15423; https://www.cytivalifesciences.com) using a Langmuir fitting model with R_max local_ (analyte binding capacity on the sensor surface). The antibody–antigen complex half-life was calculated in minutes as t_1/2_ = ln(2)/60*k_d_ (where k_d_ represents dissociation rate). The molar ratio (MR) indicating antibody–antigen binding stoichiometry was determined as MR = x/y *z_a_/z_b_ (where x is RMAX [RU], b is antibody capture level [RU], z_a_ is MW [antibody], and z_b_ is MW [analyte]).

Using the results of the Biacore analysis for the 15 tested antibodies, six (2E9, 3B10, 3H6, 13C5, 2H9, and 2C11) were selected for further analysis based on the following favourable properties: specific kinetic interaction with the fucosylated antigen PSA(67–79)-G0F, no detectable cross-reactivity with the non-fucosylated antigen PSA(67–79)-G2, functional 1:1 or 1:2 antigen-binding stoichiometry, faster association rates (k_a_), and slow dissociation rates (k_d_) (Table 1).

**Table 1:** Kinetic analyses measuring the binding of the selected (2E9, 13C5, 2C11, 2H9, 3H6, 3B10) and two of the deselected (13E12, 15F10) mAbs to PSA(67–79)-G0F.

mAb	k_a_ (M^−1^ s^−1^)	k_d_ (s^−1^)	t_1/2_ (min)	K_D_ (nM)	CL (RU)	BL (RU)	MR	Chi squared
2E9	1.75 × 10^6^	1.12 × 10^−3^	10	0.6	701	27	2	0.19
13C5	9.81 × 10^5^	3.80 × 10^−4^	31	0.4	700	29	2	0.04
2C11	7.79 × 10^5^	7.80 × 10^−4^	15	1.0	1648	53	1	0.41
2H9	5.78 × 10^5^	1.05 × 10^−3^	11	2.0	1212	54	2	0.15
3H6	1.52 × 10^6^	5.36 × 10^−3^	2	4.0	777	25	1	0.07
3B10	1.14 × 10^6^	1.21 × 10^−2^	1	11.0	897	31	1	0.07
13E12*	1.66 × 10^5^	3.56 × 10^−2^	0.3	215	758	20	1	0.54
15F10*	1.66 × 10^5^	3.99 × 10^−2^	0.3	241	649	18	1	0.44

*BL* binding late, *CL* capture level, *IHC* immunohistochemical, *k*_*a*_ association constant, *k*_*d*_ dissociation constant, *K*_*D*_ equilibrium dissociation constant, *mAb* monoclonal antibody, *min* minutes, *MR* molar ratio, *PSA* prostate-specific antigen, *RU* response unit, *t*_*1/2*_ antibody–antigen complex half-life.

*From the 15 tested antibodies, a set of 6 (indicated in bold and non-italics) was selected based on the following favourable properties: specific kinetic interaction with the fucosylated antigen PSA(67–79)-G0F; no detectable cross-reactivity with the non-fucosylated antigen PSA(67–79)-G2 (see Fig. 3); functional 1:1 or 1:2 antigen binding stoichiometry; faster association rates ka (1/Ms), and slow dissociation rates kd (1/s). Antibodies with unfavourable kinetics (e.g., 13E12, 15F10) were deselected from further evaluation.

The amino acid sequences of the V_H_ and V_L_ regions of selected antibodies with favourable kinetic characteristics were aligned using Geneious. This revealed that all six shared a surprisingly high sequence similarity in the V_H_ and V_L_ regions, particularly in the complementarity determining region (CDR). As the six mAbs displayed comparable kinetic properties and had high sequence similarity, results will be shown only for one mAb (2E9).

### Western blot analysis

Specificity of the 2E9 anti-fuc-PSA mAb was assessed by performing western blot analysis with native and deglycosylated PSA isolated from seminal fluid (Scripps Laboratories), according to published methodology that was adapted for fuc-PSA detection^33^. Additionally, western blot analysis was used to assess reactivity of the anti-fuc-PSA mAb (2E9) against other glycoproteins, including biotinylated native and deglycosylated PSA, biotinylated haptoglobin (Sigma), biotinylated AFP (Bio-Rad), and non-biotinylated apo-transferrin (R&D Systems).

### Sandwich ELISA

The applicability of the 2E9 anti-fuc-PSA mAb for sandwich ELISA was tested by combining the 2E9 with mAbs and polyclonal antibodies directed against total PSA on the Roche multimarker IMPACT (Immunological Multi-Parameter Chip Technology) platform^34^.

### Immunohistochemical assays

Immunohistochemical (IHC) assays were developed to assess the ability of the 2E9 mAb to detect fuc-PSA in formalin-fixed, paraffin-embedded (FFPE) samples of prostate adenocarcinoma. The fuc-PSA IHC assays were conducted on a VENTANA BenchMark ULTRA platform (Roche) using a VENTANA OptiView DAB IHC Detection Kit (Roche). Briefly, after deparaffinisation with EZ Prep (Roche) at 72 °C, tissue specimens were pretreated with ULTRA Cell Conditioning 1 buffer (Roche) for 80 min at 100 °C for antigen retrieval, followed by inactivation of endogenous peroxidase with hydrogen peroxide for 4 min. Specimens were incubated in Reaction buffer (Roche) with 5 µg/mL of the 2E9 mAb at 36 °C for 16 min. Following chromogenic detection, slides were counterstained with Hematoxylin II and Bluing Reagent (Roche) for 4 min each and coverslips were applied.

Stained slides were semi-quantitatively assessed by a pathologist based on staining intensity (negative staining [0], weak [1+], moderate [2+], and strong staining [3+]), and also proportion of stained tumour cells.

### Assessment of the specificity of the fuc-PSA IHC assay

To investigate the specificity of the 2E9 mAb, the staining performance of the fuc-PSA assay was evaluated on neoplastic and non-neoplastic FFPE tissue microarrays (Roche). Staining for fuc-PSA using 2E9 was compared with staining for total PSA using a commercial mAb: ER-PR8 (Roche).

To verify that the 2E9 mAb recognises and binds specifically to fuc-PSA in FFPE tissue specimens, a peptide competition analysis was performed using various synthetic peptides specific and non-specific for the target epitope. In brief, 5 µg/mL of 2E9 mAb (500 µL) was pre-incubated with varying concentrations of PSA(67–79)-G0F (500 µL) diluted in PBS, as well as its variants for 1 h at room temperature, prior to performing IHC analysis. During IHC staining on VENTANA BenchMark ULTRA system, 85 µL of these solutions (half the optimal titre of primary antibody pre-incubated with various concentrations of peptide solutions or PBS) were manually applied onto the deparaffinised and pretreated tissue sections, followed by incubation at 36 °C for 16 min. The immunolocalised fuc-PSA protein was visualised using an OptiView DAB IHC detection kit.

To further investigate the specificity of the fuc-PSA IHC assay, the N-linked glycan moieties of the PSA were removed by enzymatic deglycosylation of tissue specimens in the IHC assay. Briefly, tissue specimens were incubated with 3 units of peptide-*N*-glycosidase F (PNGase F; Roche) diluted in phosphate buffered saline (PBS), or PBS alone (control), for 32, 60, and 120 min at 36 °C prior to comparing IHC staining with the 2E9 mAb and commercial anti-total PSA mAb (ER-PR8).

### X-ray crystallography

Detailed structural analyses were performed using X-ray crystallography to explore the interaction between fuc-PSA and the cloned 2E9 anti-fuc-PSA mAb.

To obtain the apo fragment antigen-binding region (Fab) structure, crystallisation screening was performed using a solution containing the 2E9 Fab fragment concentrated to 18 mg/mL in 20 mM HEPES pH 7.5 and 100 mM NaCl. To obtain a structure of the fuc-PSA peptide-bound Fab, the Fab was concentrated to 20 mg/mL with 5 M excess peptide. Crystallisation droplets were set up at 21 °C by mixing 100 nL of protein solution with 100 nL of reservoir solution (1:1), or 140 nL of protein solution with 60 nL of reservoir solution (7:3) in a vapour diffusion sitting drop experiment.

Crystals used for structure determination of the apo form appeared after 3 days and grew to full size within 21 days in the following precipitant solution: 100 mM potassium thiocyanate and 30% w/v PEG MME 2000 (poly[ethylene glycol] methyl ether). Crystals containing the peptide-bound form appeared and grew to full size within 2 days in the following precipitant solution: 100 mM Tris-HCl pH 8.5, 6.25% v/v PEG 3350, 6.25% v/v PEG 4000, 6.25% v/v PEG 2000 and 6.25% v/v PEG 5000 MME.

Apo crystals were harvested from 7:3 droplets and peptide-bound crystals were harvested from 1:1 droplets. Both crystal forms were soaked for 5 min in the crystallisation buffer containing additional 20% v/v ethylene glycol before flash-cooling in liquid nitrogen.

Diffraction images were collected with an EIGER2X 16 M detector at a temperature of 100 K at the beam line X10SA of the Swiss Light Source and images were processed with the XDS package^35^. For the apo crystal, data from a single crystal were merged to yield a 2.25 Å resolution data set in space group P1211 with two molecules in the asymmetric unit (Supplementary Table 1). For the peptide-bound crystal, similarly, data from a single crystal were merged to yield a 1.38 Å resolution data set in space group P212121 with two molecules in the asymmetric unit (Supplementary Table 1).

The structure was determined by molecular replacement with the program PHASER^36^ as a part of the PHENIX suite^37^. A Fab fragment (protein databank [PDB] accession code 4ZTP) was split into constant and variable domains, which were used as search models. The molecular replacement solution model was rebuilt in COOT^38^ and refined with PHENIX Refine. An analysis by the program PISA^39^ was used to characterise and define the ligand binding.

## Results

### Immunisation strategy

The polyclonal sera from all immunised rabbits bound to the PSA(67–79)-G0F screening peptide, a glycopeptide consisting of amino acids 67–79 of PSA containing the Asn-69 *N*-glycosylation with an octasaccharide linked to a core α-1,6-fucose, showed positive specific signal on ELISA testing. Thus, all animals were suitable for subsequent antibody development using the B-cell cloning method.

Screening identified 16 clones that could bind to PSA(67–79)-G0F and discriminate from the negative screening agents according to the selected cut-offs (OD > 0.6 in positive screening and < 0.6 in negative screening). These included PSA(67–79)-G2, a glycopeptide with the same peptide sequence as PSA(67–79)-G0F and containing an Asn-69 glycan with a nonasaccharide but lacking the core α-1,6-fucose; and Asn-G0F, an octasaccharide with a core α-1,6-fucose (representing glycan without the PSA peptide) bound to an Asn residue. The results of the screening thereby demonstrated that both protein and carbohydrate moieties of PSA were required for mAb binding. Of the 16 clones, 15 successfully underwent molecular cloning and V_H_ and V_L_ domain sequence determination. The V_H_ and V_L_ domain sequence for the 2E9 mAb are presented in Supplementary Fig. 1 Ref.^40^.

### Antibody characterisation

#### Western blot analysis

In an SDS-PAGE (sodium dodecyl sulphate–polyacrylamide gel electrophoresis) western blot analysis, the 2E9 mAb was reactive for and, thus, could identify, the native PSA (Supplementary Fig. 2A). Contrastingly, no reactivity was observed to deglycosylated native PSA (Supplementary Fig. 2B

). Additionally, the 2E9 mAb did not react with other proteins carrying similar core-fucosylated glycostructures, including haptoglobin (which has four N-linked glycosylation motifs), AFP (one N-linked glycosylation motif) or transferrin (three N-linked and one O-linked glycosylation motifs) (Supplementary Fig. 3b–f) Ref.^41^. In loading-control western blot with streptavidin-POD (peroxidase), the biotin-labelled proteins were all detected; Supplementary Fig. 3h–k), while transferrin (non-biotinylated) was not (Supplementary Fig. 3l).

#### Sandwich ELISA

The most reactive immunological sandwich was formed by the combination of capture rabbit polyclonal anti-total PSA antibodies K-54794 (Novus) with the anti-fuc-PSA mAb 2E9 for detection.

Sample pre-treatment with 100 mM Tris (pH 12.9), 30.6 mM TCEP (Tris [2-carboxyethyl] phosphine) and 2 mM EDTA resulting in protein reduction was necessary to allow reactivity of the 2E9 mAb to linearised PSA. Under these conditions, all sandwich assays reacted with purified seminal PSA antigen spiked in artificial serum matrix, but not with corresponding concentrations of deglycosylated PSA, nor unrelated glycoprotein CD59 (Supplementary Fig. 4).

#### Specificity of the fuc-PSA IHC assay

The anti-fuc-PSA mAb (2E9) demonstrated strong specific staining in prostate tissue samples. Only focal weak cytoplasmic staining was detected in kidney (cortical tubules), liver (hepatocytes), lung (pneumocytes), nerve and liposarcoma tissue specimens (Supplementary Tables 2, 3).

IHC analyses demonstrated a similar staining pattern in FFPE prostate tissue adenocarcinoma specimens for fuc-PSA (assessed with anti-fuc-PSA mAb 2E9) and total PSA (assessed with the commercially available mAb, ER-PR8, Roche), with slightly weaker staining intensity and less coverage for fuc-PSA (Supplementary Fig. 5). The secretory epithelial cells of the prostate glands displayed a moderate-to-strong cytoplasmic staining of fuc-PSA, with more intense staining in the apical portion of the prostatic epithelium.

**Figure 1:**
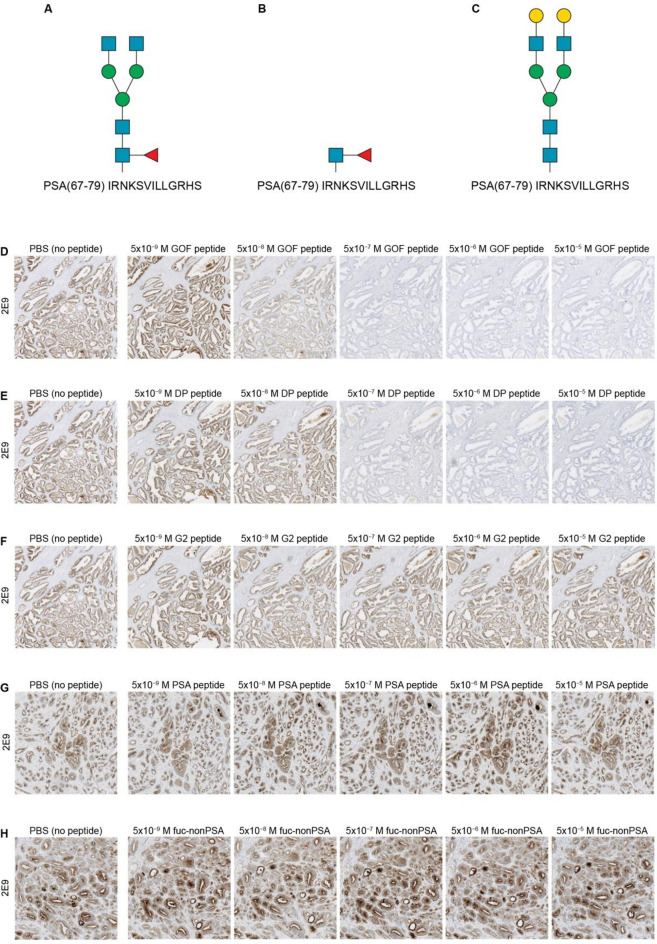
Peptide inhibition analysis with synthetic peptides. This figure shows a schematic representation of glycans according to Symbol Nomenclature For Glycans (SNFG) hosted by NCBI, National Center for Biotechnology Information ^44,45^. Peptides included in the analysis were (**A**) PSA(67–79)-G0F, (**B**) fucosylated PSA disaccharide glycopeptide, PSA(67–79)-DP, and (**C**) PSA(67–79)-G2. IHC analysis of FFPE samples of prostate adenocarcinoma samples showed inhibition of binding of the fuc-PSA mAb (2E9) by (**D**) PSA(67–79)-G0F and (**E**) PSA(67–79)-DP. No inhibition of binding of the fuc-PSA mAb (2E9) was seen with (**F**) non-fucosylated PSA glycopeptide (PSA(67–79)-G2), (**G**) aglycosylated PSA fragment (aa 67–79) or (**H**) an α-1,6-fucosylated non-PSA octasaccharide glycopeptide (fuc-nonPSA). In **D–H**, 2E9 fuc-PSA antibodies at 2.5 µg/mL were pre-incubated with PBS or varying concentrations of peptide (as indicated). FFPE, formalin-fixed, paraffin-embedded; fuc-PSA, α-1,6-fucosylated PSA; IHC, immunohistochemical; PBS, phosphate buffered saline; PSA, prostate-specific antigen.

In peptide competition analysis, fuc-PSA staining decreased following pre-incubation with increasing molar ratios of fuc-PSA(67–79)-G0F or disaccharide glycopeptides, fuc-PSA(67–79)-DP (Fig. 1). This indicates that these peptides bound competitively to anti-fuc-PSA mAb 2E9 and inhibited target epitope binding in the IHC assay. By contrast, no inhibition to the binding of the 2E9 mAb to fuc-PSA was observed when the 2E9 mAb was pre-incubated with at least 100-fold excess (5 × 10^−5^ M) of non-fucosylated antigen (PSA[67–79]-G2), the aglycosylated PSA fragment (aa 67–79), or an α-1,6-fucosylated non-PSA octasaccharide glycopeptide.

**Figure 2:**
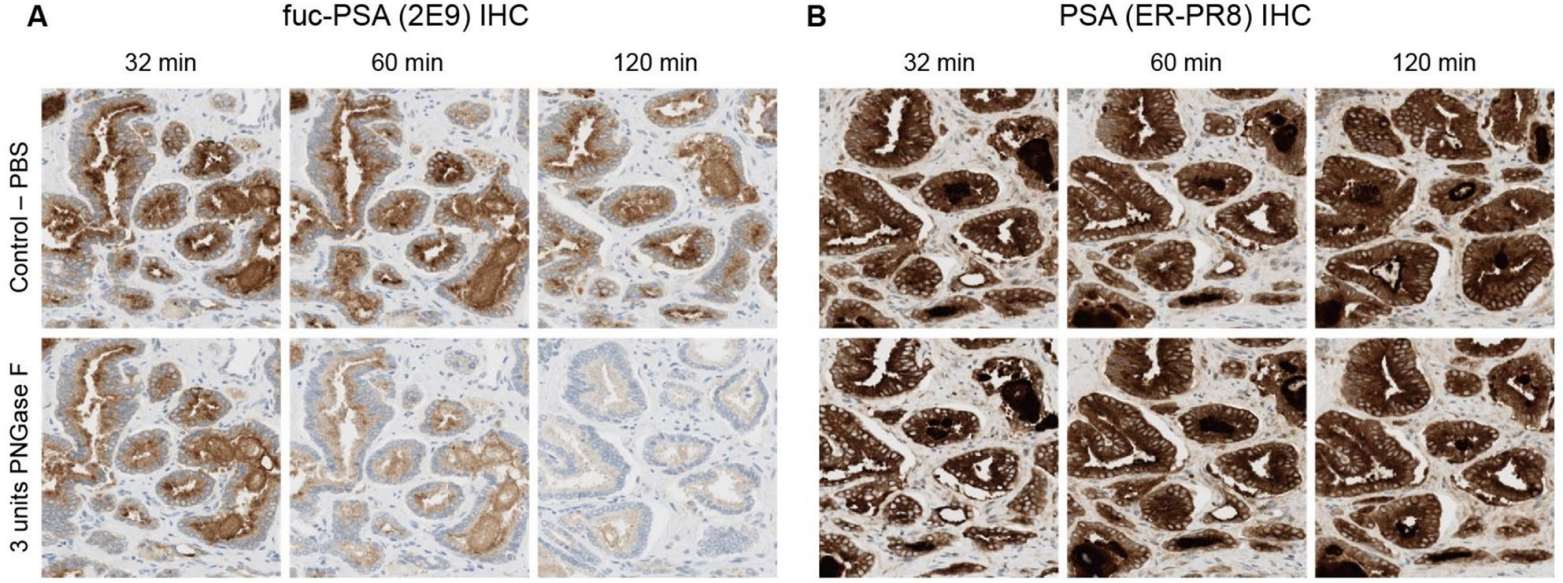
Deglycosylation of fuc-PSA. IHC analysis of FFPE prostate adenocarcinoma samples showing (**A**) substantially reduced fuc-PSA (2E9) immunostaining with increased PNGase F treatment incubation time, and (**B**) no changes in total PSA detection. Prior to primary antibody incubation, tissue specimens were treated with, top to bottom rows, PBS buffer (control) or 3 units of PNGase F diluted in PBS buffer for 32, 60 and 120 min. Samples were then immunostained with (**A**) fuc-PSA (2E9) or (**B**) PSA (ER-PR8) IHC assays. FFPE, formalin-fixed, paraffin-embedded; fuc-PSA, α-1,6-fucosylated PSA; IHC, immunohistochemical; PBS, phosphate buffered saline; PSA, prostate-specific antigen.

Treatment of tissue specimens with PNGase F to remove global N-linked glycosylation resulted in substantially reduced anti-fuc-PSA mAb (2E9) immunostaining compared with non-glycosylated tissue specimens (incubated with PBS), while no changes were observed in total PSA detection using commercial mAb between deglycosylated and native tissue samples (Fig. 2).

#### X-ray crystallography

**Figure 3:**
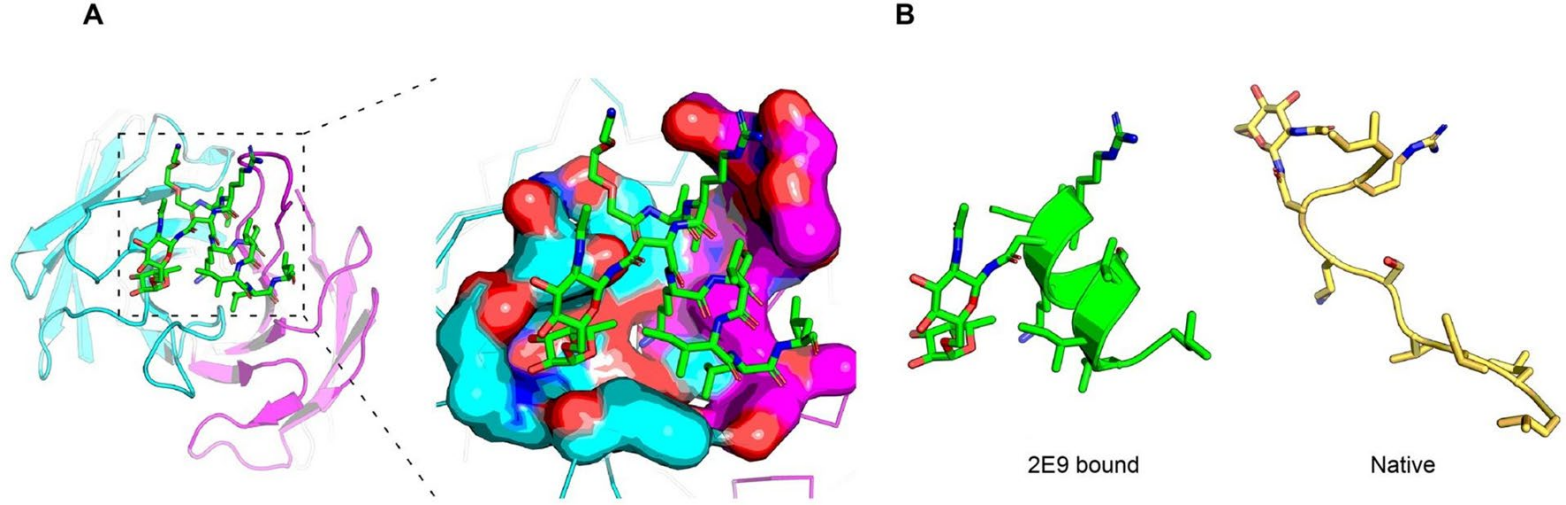
Crystal structure of 2E9 bound to PSA disaccharide glycopeptide. (**A**) Overview of the antibody and peptide interaction, with the V_H_ shown in cyan blue, V_L_ shown in magenta, and peptide shown in green. The peptide forms a short α-helix that fits snuggly in a central pocket between the V_H_ and V_L_. The long CDR 3 loops of each chain encapsulate the peptide and the disaccharide in a single large pocket. The inset image shows a surface rendering of the antibody, indicating the shape and size of the binding pocket. (**B**) The fuc-PSA peptide adopts an α-helical form when bound by the 2E9 anti-fuc-PSA mAb, as shown in green (left), whereas the same region in the native protein, shown in yellow, is linear and not twisted (right). CDR, complementarity determining region; fuc-PSA, α-1,6-fucosylated PSA; PSA, prostate-specific antigen; V_H_, variable-domain heavy chain; V_L_, variable-domain light chain.

The crystal structure of the 2E9 mAb bound to a fuc-PSA peptide reveals an extensive paratope that includes 29 residues and a surface area of 760 Å (Fig. 3). The large pocket binds nine residues of the peptide, spanning Ile67–Leu75, and the N-linked glycosylation site containing two sugar moieties, N-acetylglucosamine (GlcNAc) and fucose. The sugars are exclusively bound by the heavy chain via polar/charge-based interactions, while the peptide amino acid residues are bound by both the heavy and the light chain via a mixture of polar/charge and hydrophobic interactions, with the latter predominately arising from the light chain (Supplementary Fig. 6). Notably, the bound peptide adopts an α-helical form when bound by the 2E9 mAb, whereas in the context of the entire PSA protein, the peptide adopts a linear form. Thus, a twist of the peptide is required upon binding which in the context of the intact protein is not possible without unfolding. In silico modelling was used to explore the binding of 2E9 to a full N-glycan structure, representative of those typically found on PSA. This was achieved by adding four sugar moieties to the ligand *in-silico* to represent the core of a branching glycan tree (Supplementary Fig. 7). The simple model showed that the 2E9 mAb can bind to any complex branching glycan, as none of the core sugar moieties beyond those crystallised were seen to sterically clash with the antibody.

Comparison between the bound and the apo structures of 2E9 shows a mobile light chain CDR 3 loop. The loop opens the pocket in the apo state and may act as a type of lid that closes upon peptide binding. Other minor conformational changes occur in the heavy chain CDR 2 loop, but other parts of the pocket remain the same between the two structures.

## Discussion

Antibodies that recognise both the glycan and protein moieties of glycoproteins offer the potential to advance current glycobiology techniques, facilitating the development of improved immunological, antibody-based glycosylation detection. Here, we have described the development of a novel mAb that specifically recognises fuc-PSA.

Generation of high-affinity, glycan-specific antibodies has traditionally been viewed as challenging because of the low immunogenicity of carbohydrate moieties and because glycan modifications, such as α-1,6-fucose, are common in animals used for antibody development^17,24^. In the current study, immunogens representing the core-fucosylated form of PSA were generated and immunisation with these peptides resulted in the production of individual IgG clones that reacted specifically with a fuc-PSA glycopeptide (PSA[67–79]-G0F), but not with the glycopeptide without the core-fucose residue (PSA[67–79]-G2). Notably, non-binding to the Asn-G0F was also screened with ELISA. The results therefore demonstrate that the mAbs expressed from the six selected clones, including 2E9, are highly specific and detect the α-1,6-fucosylated form of PSA, but not the aglycosylated PSA or glycan without the PSA peptide. We also established that the 2E9 mAb did not bind to non-PSA glycoproteins with similar core fucosylated N-glycan (i.e., haptoglobin, AFP, or transferrin).

The immunisation approach used may be more broadly applicable and may represent a means of generating novel antibodies to other glycoprotein targets beyond prostate cancer biomarkers. A similar approach has been adopted to develop antibodies targeting the *Lens culinaris* agglutinin-reactive fraction of α-fetoprotein, which is specifically increased in patients with HCC^17^.

The antibodies generated were extensively characterised and demonstrated a high specificity and affinity in kinetic analysis using a fuc-PSA peptide. Results of western blot analyses confirmed specificity of the identified antibodies for core-fucosylated PSA (PSA[67–79]-G0F), and also selectivity for PSA(67–79)-G0F over the non-core fucosylated-PSA (PSA[67–79]-G2). An IHC assay to detect fuc-PSA protein expression in FFPE prostate tissue specimens was developed using the anti-fuc-PSA mAb 2E9. Specificity of the fuc-PSA (2E9) IHC assay was further evaluated through primary antibody peptide inhibition and deglycosylation analysis. The data from these analyses collectively demonstrate that the fuc-PSA (2E9) IHC assay has high specificity and only detects fuc-PSA protein in FFPE prostate tissue specimens.

Inhibition of antibody binding by both octa- and disaccharide fuc-PSA peptides indicates that the 2E9 mAb can bind the majority of the α-1,6-fucosylated PSA glycoforms, as the fuc-GlcNAc motif is conserved in core-fucosylated PSA moieties. Since chromogenic IHC is a semi-quantitative method, we were unable to determine the percentage of fucosylated PSA that was present in human tissue. However, as MS-based data indicate stable expression of fuc-PSA in up to 70–85% in seminal fluid^42^, human serum^1^ and urine^43^, it may be that a similar percentage is found in tissue.

The mode of interaction between the anti-glycopeptide antibody (2E9) and fuc-PSA was evaluated with detailed structural analyses of antibody–ligand complexes using X-ray crystallography. This showed that the 2E9 mAb binds to an α-helical form of the glycopeptide, whereas the PSA epitope is linear in the natively folded protein, thus showing that twisting or refolding of the peptide is needed for Fab binding to occur. Structural analysis of a binding complex provides significant information that not only confirms other data, such as kinetic characterisation, but can also reveal unforeseen results, such as the ligand conformation. Structural characterisation is currently the only tool, which provides both chemical and spatial mapping of an epitope and is an under-utilised tool in the development of diagnostic assays. In this study, the crystal structure was crucial in defining the application scope and helped to explain why no binding to the untreated native protein could be observed. In this context, it delivered valuable information on the optimal assay conditions and directions for successful diagnostic assay development. Furthermore, to the best of our knowledge, this is the first structure of an antibody that shows specificity to a glycosylated peptide.

In silico modelling showed that none of the core sugar moieties in a more complex native N-glycan structure, beyond those crystallised in the disaccharide structure, sterically clash with the 2E9 mAb. Furthermore, this model does not consider the inherent flexibility of glycans, which would most likely increase the accessibility of the glycan to the 2E9 mAb.

There is an unmet need for prostate cancer biomarkers, as the current PSA test has poor sensitivity and specificity. PSA is not a prostate cancer-specific biomarker and analyses of blood levels are unable to discriminate between prostate cancer and other prostatic diseases, including benign prostatic hyperplasia or prostatitis^1,7,8^. Thus, widespread use of this assay results in significant overdiagnosis and overtreatment of men with indolent disease^3,5,6^.

Several research groups have postulated that analyses of altered glycosylation could increase the diagnostic potential of PSA^11^. Assessment of changes in the degree of PSA fucosylation in the sera of cancer patients have been described using different lectin-based and MS-based approaches^4,5,20^, but reported results are contradictory and thus non-conclusive. No information regarding the differences in glycosylation status of PSA in PCa and normal prostate tissue has been reported so far.

The development of antibodies specific to a glycosylated protein is noteworthy as it provides opportunities for antibody-based glycosylation detection and represents a novel approach for the detection of this type of post-translational modification. An antibody-based technique to detect glycosylated PSA offers advantages over other approaches including the possibility to detect/quantify specific glycosylation patterns, which is generally not possible with lectin-based approaches because of poor sensitivity and specificity^1,9,24^. An antibody-based approach provides a robust method to investigate glycosylation status of PSA in healthy and diseased samples. Antibodies may also represent a more straightforward and inexpensive method for glycosylation analyses, and may provide better analytical sensitivity, compared with MS techniques, which can have prolonged measuring times and are generally complex to perform^1,6^. Antibody-based assays also offer the potential for an automated, high-throughput approach.

The application of the assay described here is currently optimised for tissue samples, as sandwich ELISAs showed relatively weak mAb binding to PSA in serum samples, and sample pre-treatment (reduction and/or denaturation of PSA) was required for specific detection, in agreement with the crystal structure of the antibody–ligand complex. The development of an antibody-based sandwich ELISA assay for clinical application will require further investigation.

In terms of next steps, it will be important to understand the role of an altered PSA glycosylation pattern in the development of prostate cancer and its diagnostic or therapeutic potential, as there is currently a lack of consensus on this topic. It will also be helpful to understand the utility of combining an antibody-based assay with other modalities (e.g., imaging or serum biomarker testing). Epitope characterisation by structural methods will continue to play a crucial role in validating and rationalising results from kinetic analysis on specificity and accuracy. This is currently the best approach to obtain precise information regarding an antibody’s epitope, but it also provides other significant information, such as the ligand spatial and chemical conformation, that cannot be elucidated by other methods.

This research introduces a well-characterised, first-in-class antibody capable of binding fuc-PSA and presents the first crystal structure of an antibody demonstrating glycosylation-specific binding to a glycopeptide.

### Supplementary Information

Supplementary Information.

## Data Availability

Crystallographic data has been deposited in the Protein Data Bank (www.pdb.org; PDB IDs 9F18 and 9F1I).
