# Supplementary material for: Development of a first-in-class antibody and a specific assay for α-1,6-fucosylated prostate-specific antigen
Source: Sci Rep. 2024 Jul 17;14:16512. doi: 10.1038/s41598-024-67545-1 (PMC11254934; doi:10.1038/s41598-024-67545-1)
Supplement: Supplementary file 1 — Supplementary Information. [file 41598_2024_67545_MOESM1_ESM.pdf]

Supplementary Information

Title: Development of a first-in-class antibody and a specific assay for α-1,6-fucosylated prostate-specific antigen

Authors:

Steinar Halldórsson[1], Lars Hillringhaus[2], Caroline Hojer[2], Andrea Muranyi[3], Michael Schraeml[2], Magdalena Swiatek-de Lange[2]*, Gloria Tabarés[2]

Affiliations:

[1]F. Hoffmann-La Roche AG, Pharma Research and Early Development, Basel, Switzerland

[2]Roche Diagnostics GmbH, Nonnenwald, Penzberg, Germany

[3]Roche Diagnostics Solutions, Tucson, AZ, USA

*Corresponding author

email address: magdalena.swiatek-de_lange@roche.com

Supplementary Figure 1. Sequence of the (**A**) V_H and (**B**) V_L domain of the 2E9 mAb[1]

A.

QSLEESGGDL	VKPGASLTLT	CTASGFSFSA	GYDMCWVRQA	PGKGLEWIAC	IYADGSGSTY	60
YANWAKGRFT	ISLASSTTVT	LQMTGLTAAD	TATYFCAREG	ADGPDYGYAA	FSLWGPGTLV	120
TVSSGQPKAP	SVFPLAPCCG	DTPSSTVTLG	CLVKGYLPEP	VTVTWNSGTL	TNGVRTFPSV	180
RQSSGLYSLS	SVVSVTSSSQ	PVTCNVAHPA	TNTKVDKTVA	PSTCSKPTCP		230

B.

DVVMTQTPAS	VEAAVGGTVT	IKCQASQSIS	NYFSWYQQKP	GQPPKLLIYK	ASTLASGVPS	60
RFKGSGSGTE	FTLTISDLEC	ADAATYYCQS	FYGSVTSDYG	GFAFGGGTEV	VVKGDPVAPT	120
VLIFPPAADQ	VATGTVTIVC	VANKYFPDVT	VTWEVDGTTQ	TTGIENSKTP	QNSADCTYNL	180
SSTLTLTSTQ	YNSHKEYTCK	VTQGTTSVVQ	SFNRGDC			217

mAb, monoclonal antibody; V_H, variable-domain heavy chain; V_L, variable-domain light chain.
[1] Swiatek-de Lange, M. et al. Antibodies specific for α-1,6-core-fucosylated PSA and fucosylated fragments thereof. International patent application no. PCT/EP2021/075964. Date of filing: 21 September 2021.

Supplementary Figure 2. Reactivity of anti-fuc-PSA mAb (2E9) in a western blot analysis showing:

(A). Reactivity with decreasing concentrations of native PSA purified from seminal fluid (lanes b to e) and biotinylated native PSA (lanes f to i); lanes (b) and (f) = 5 µg, lanes (c) and (g) = 2.5 µg, lanes (d) and (h) = 1 µg, and lanes (e) and (i) = 0.5 µg; lanes (a) and (j) contain western blot standards.

(B). Reactivity with native PSA from seminal fluid and deglycosylated native PSA. Line (c) and (f) 5 µg and 10 µg of native PSA, respectively. Line (d) and (g) 5 µg and 10 µg of deglycosylated PSA, respectively. Control lane (e) includes only the deglycosylation enzyme PNGase F. Observed smear in lane (g) is caused by the presence of PNGase F in highly concentrated deglycosylated sample and by the overexposure of the membrane, similarly to control lane (e). Lines (a), (b), (i) and (j) contain western blot standards.

A.

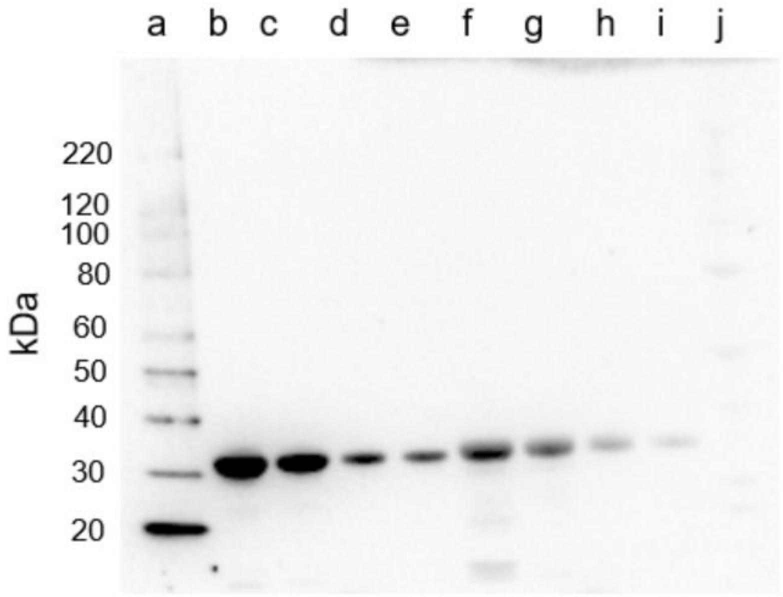

B.

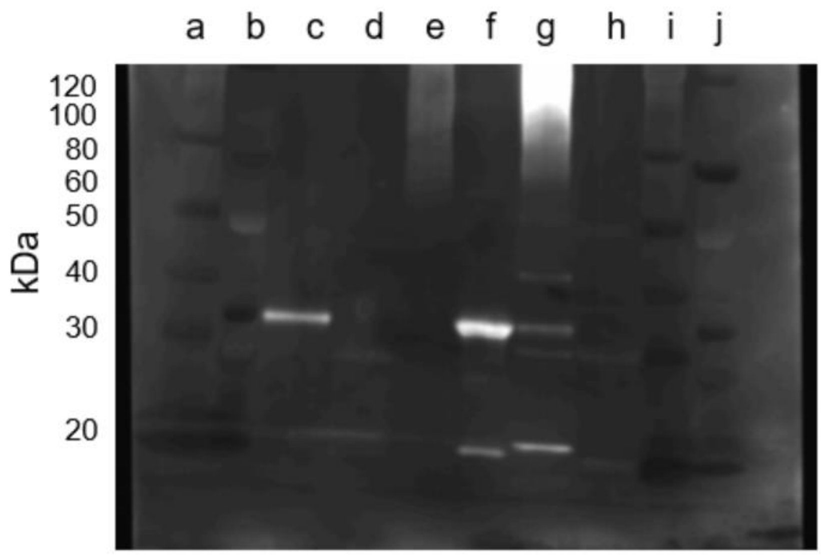

fuc-PSA, α-1,6-fucosylated PSA; PSA, prostate-specific antigen; PNGase F, Peptide N-Glycosidase F.

Supplementary Figure 3. Western blot analysis showing reactivity of anti-fuc-PSA mAb (2E9) against biotinylated native PSA (b), deglycosylated biotinylated PSA (c), biotinylated haptoglobin (d), biotinylated AFP (e), and apo-transferrin (f). Reactivity of streptavidin-POD against biotinylated native PSA (h), deglycosylated biotinylated PSA (i), biotinylated haptoglobin (j), biotinylated AFP (k), and apo-transferrin (l); Lanes (a) and (g) contain western blot standards.

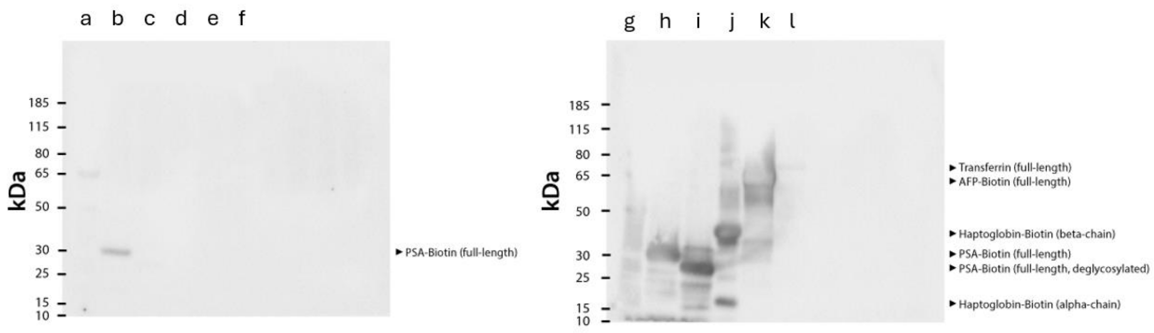

AFP, α-fetoprotein; mAb, monoclonal antibody; POD, peroxidase; PSA; PSA, prostate-specific antigen.

Supplementary Figure 4. Sandwich ELISA analysis using anti-total PSA antibody K-54794 (capture antibody) and anti-fuc-PSA mAb, (2E9, detection antibody) with native (diamonds) and deglycosylated (squares) seminal fluid PSA antigen after spiking it in artificial serum matrix.

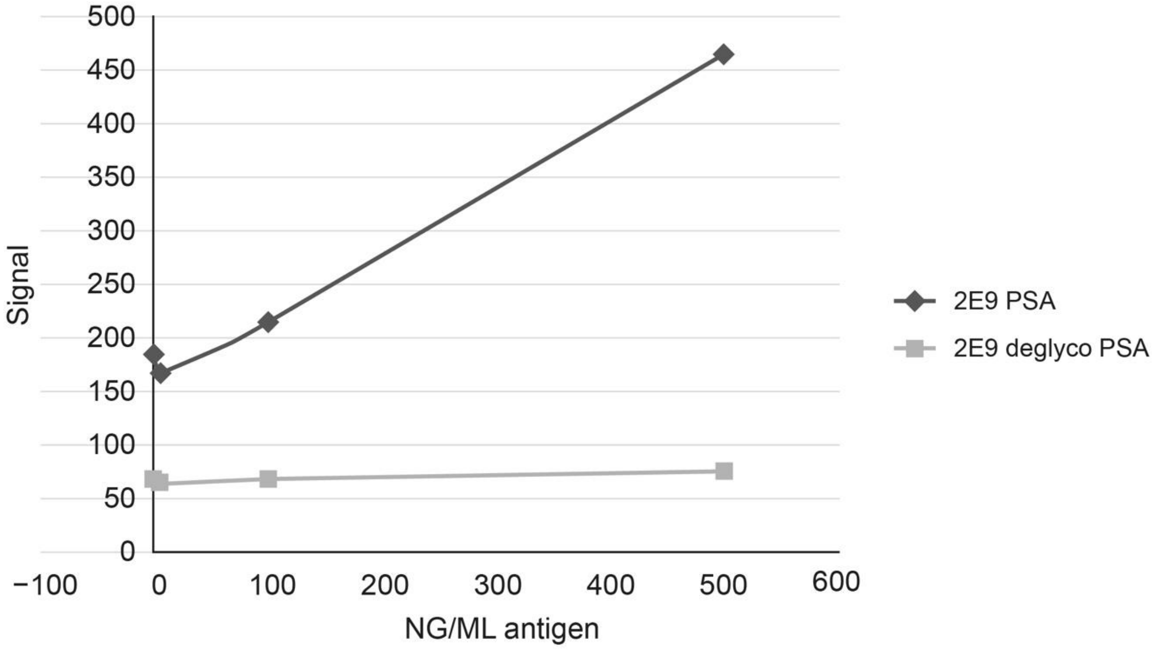

fuc-PSA, α-1,6-fucosylated PSA; mAb, monoclonal antibody; PSA, prostate-specific antigen.

Supplementary Figure 5. IHC staining of (**A**) core-fucosylated PSA as detected using anti-fuc-PSA mAb (2E9) and (**B**) total PSA in FFPE biopsies of prostate adenocarcinoma as detected using commercially available anti-PSA mouse mAb (ER-PR8, Roche).

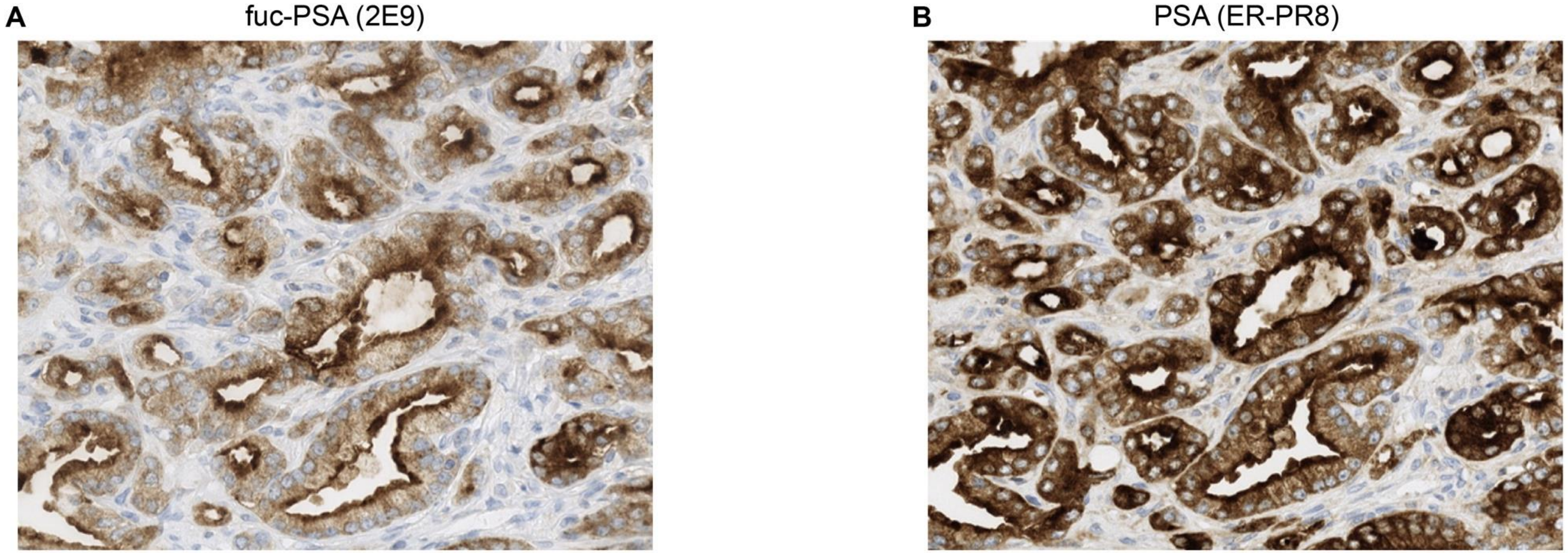

FFPE, formalin-fixed, paraffin-embedded; fuc-PSA, α-1,6-fucosylated PSA; IHC, immunohistochemical; mAb, monoclonal antibody; PSA, prostate-specific antigen.

Supplementary Figure 6. Additional crystal structure details of 2E9 bound to PSA disaccharide glycopeptide. Overview of the antibody and peptide interaction, with the V_H shown in cyan blue, V_L shown in magenta, and peptide shown in green. The binding pocket is shown in stick representation, and polar and hydrogen bonds are displayed as dashed lines. Key 2E9 mAb residues that interface and interact with the ligand are annotated. (**A**) Most of the mAb-sugar interactions are polar/charge based, while interactions with (**B**) the peptide are a mixture of polar/charge and hydrophobic interactions, with many of the hydrophobic interactions arising from the light chain. (**C**) A stereo-image of the antibody and peptide interaction

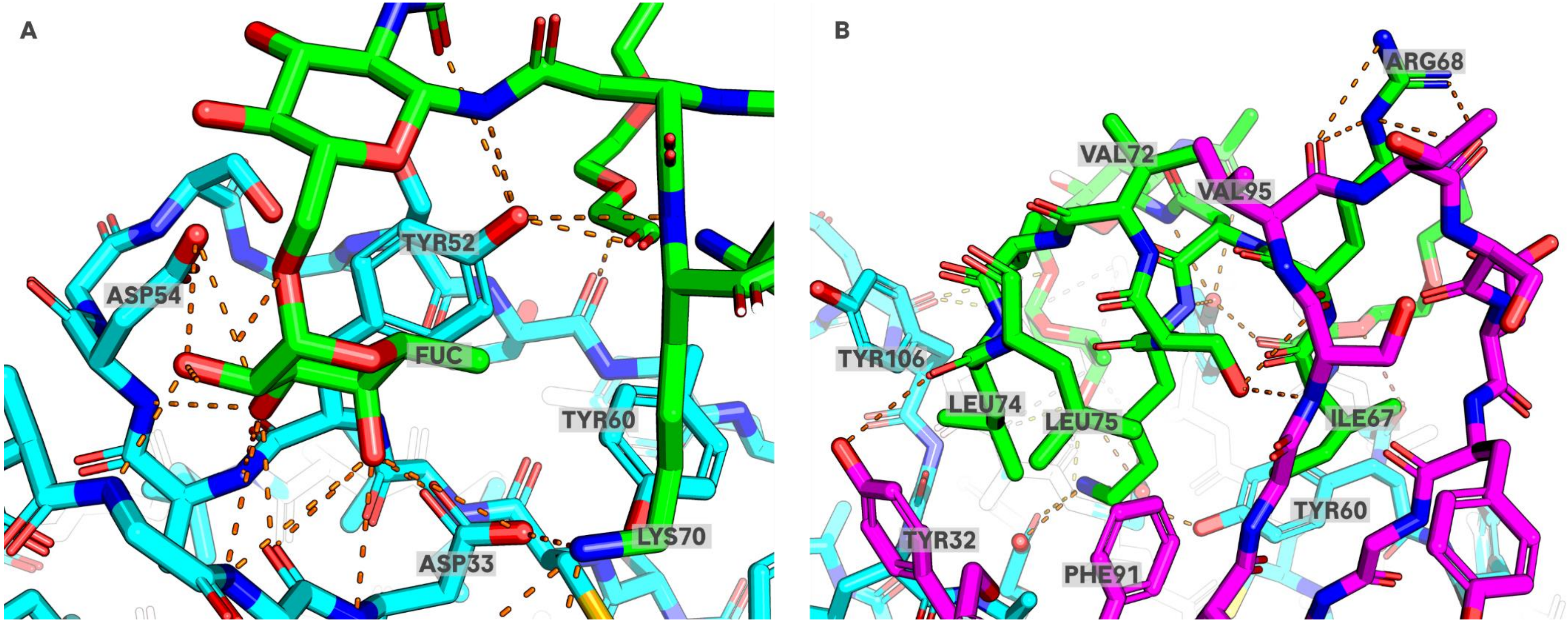

C

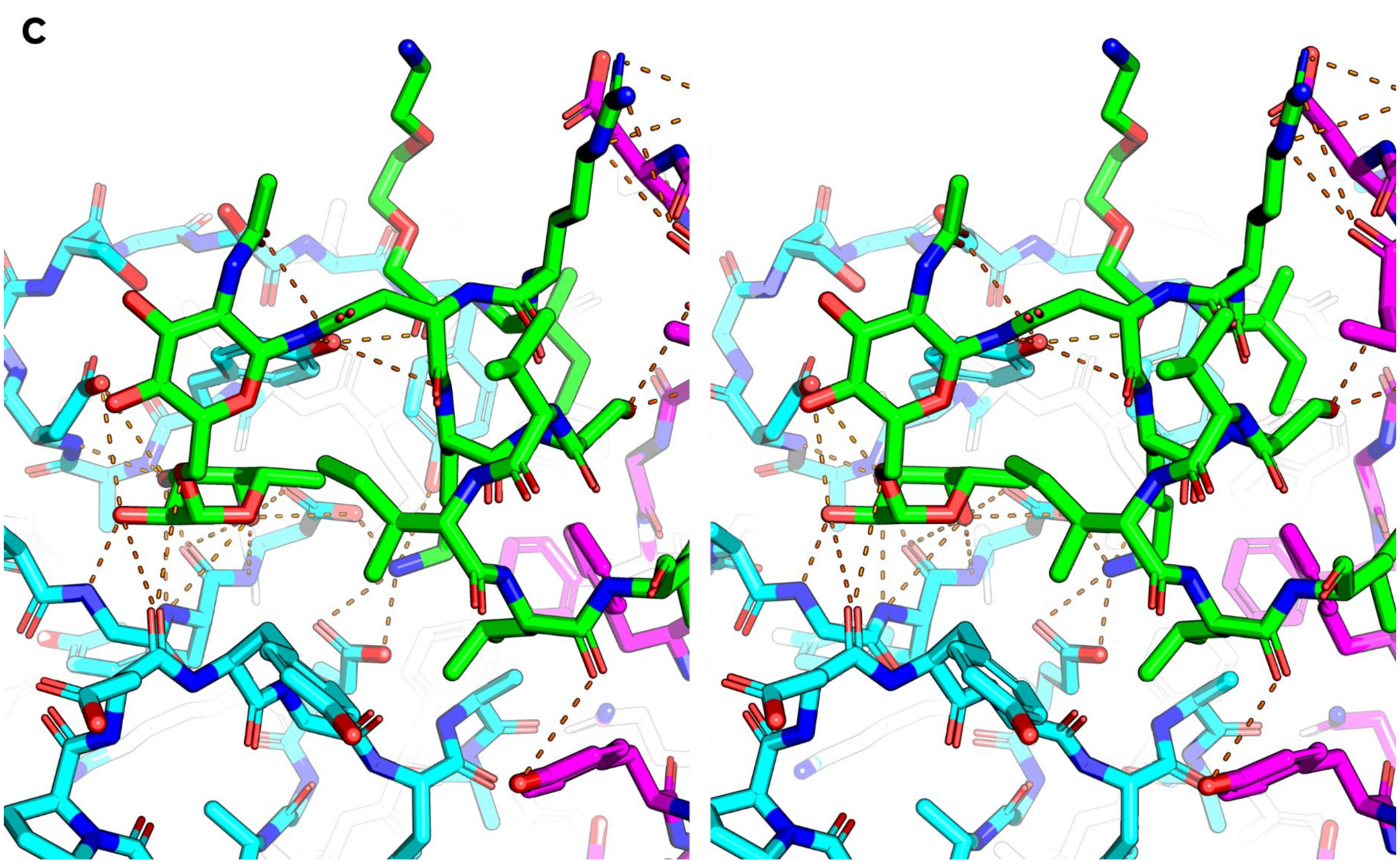

PSA, prostate-specific antigen; V_H, variable-domain heavy chain; V_L, variable-domain light chain.

Supplementary Figure 7. (**A**) Crystal structure of 2E9 bound to PSA disaccharide glycopeptide with an additional four sugar moieties built onto the existing N-linked glycosylation site to generate a core glycan that is common with most mammalian glycosylation: alpha-D-mannopyranose-(1-6)-[alpha-D-mannopyranose-(1-3)]beta-D-mannopyranose-(1-4)-2-acetamido-2-deoxy-beta-D-glucopyranose-(1-4)-[alpha-L-fucopyranose-(1-6)]2-acetamido-2-deoxy-beta-D-glucopyranose. The V_H is shown in cyan blue, V_L shown in magenta, peptide shown in green, and the additional four sugar moieties shown in pale green. (**B**) A schematic diagram of the glycan core, including linkage information. N-acetylglucosamine is represented by a blue square, fucose by a red triangle, and mannose by a green circle. The four additional sugar moieties are highlighted within the dotted frame.

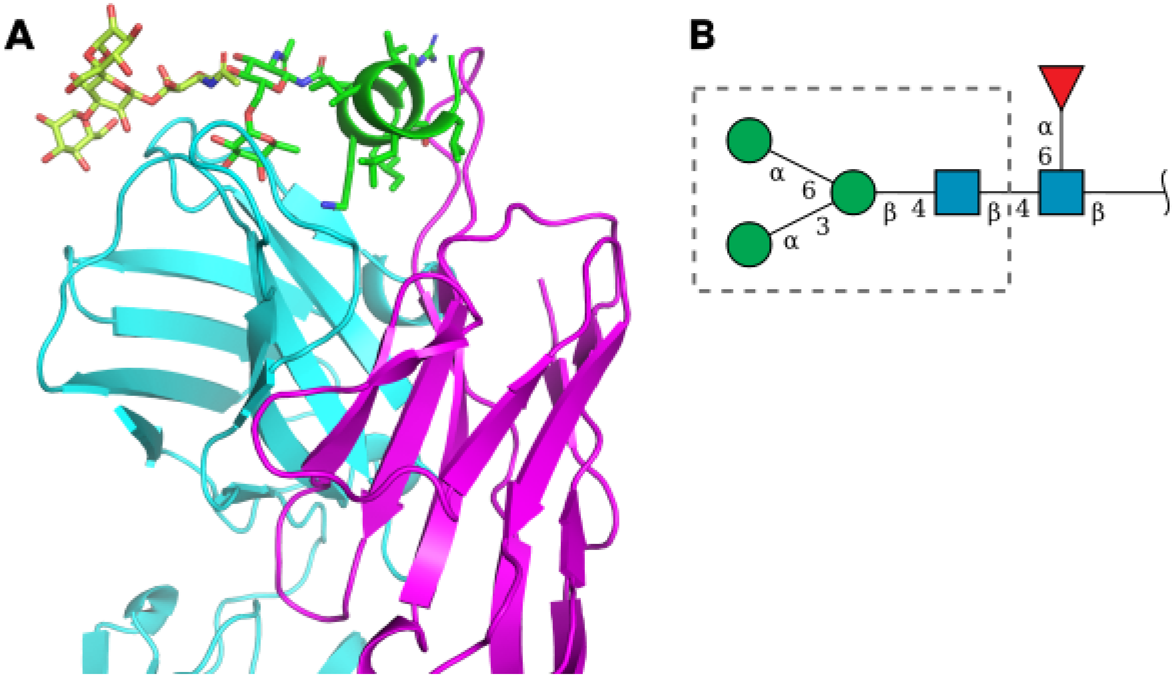

PSA, prostate-specific antigen; V_H, variable-domain heavy chain; V_L, variable-domain light chain.

Supplementary Table 1. X-ray crystallography data collection parameters and refinement statistics.

	**Apo crystal**	**Anti-fuc-PSA bound crystal**
Wavelength (Å)	1.0	1.0
Resolution (Å)	66–2.53 (2.80–2.53)	52.24–1.38 (1.49–1.38)
Spacegroup	P1211	P212121
Unit cell dimensions<br>a,b,c (Å)<br>α,β,γ (°)	72.22, 66.74, 91.71<br>90.00, 112.09, 90.00	72.31, 87.40, 150.43<br>90.00, 90,00, 90.00
Total reflections	59,928 (2,831)	1,050,559 (56,755)
Unique reflections	19,541 (978)	155,757 (7,791)
Multiplicity	3.1 (2.9)	6.7 (7.3)
Completeness (%)	90.5 (50.7)	95.7 (65.4)
Mean I/σ(I)	15.2 (1.4)	15.7 (1.5)
Wilson B-factor	70.84	27.86
R-meas	0.087 (1.089)	0.054 (1.169)
CC1/2	0.999 (0.503)	0.999 (0.633)
**Refinement**		
Reflections used in refinement	18,106 (1366)	147,987 (6027)
Reflections used for R-free	901 (74)	7456 (307)
R-work	18.87	15.52

	**Apo crystal**	**Anti-fuc-PSA bound crystal**
R-free	23.70	18.31
Number of non-hydrogen atoms	6,459	7,511
Macromolecules	6,456	6,622
Protein residues	892	910
RMS bonds (Å)	0.002	0.008
RMS angles (°)	0.495	0.946
Ramachandran favoured (%)	96.53	98.19
Ramachandran allowed (%)	3.47	1.81
Ramachandran outliers (%)	0.00	0.00
Rotamer outliers (%)	1.52	0.81
Clashscore	5.21	0.91
Average B-factor ($Å^2$)	81.28	22.97
Macromolecules	81.28	21.84
Ligands	NA	23.99
Solvent	41.40	32.22

Data in parenthesis indicate the highest resolution shell.

fuc-PSA, α-1,6-fucosylated PSA; NA, not available; RMS, root mean square.

Supplementary Table 2. IHC staining analyses with anti-fuc-PSA mAb (2E9) on neoplastic tissue microarray.

Core ID	Tissue type	Diagnosis	Cancer classification	Stain intensity	Background	Comments
A1	Adrenal gland	Bening tumour	N/A	0	0	N/A
A2	Adrenal gland	Low-grade neoplasia with malignant potential	N/A	0	0.25	N/A
A3	Appendix	Low-grade neoplasia with malignant potential	N/A	0	0	N/A
A4	Bladder	Malignant primary	Carcinoma	0	0	N/A
A5	Bladder	Malignant primary	Carcinoma	0	0	N/A
A6	Lymph node	Malignant primary	N/A	0	0	N/A
A7	Breast	In situ	Carcinoma	0	0	N/A
A8	Breast	Malignant primary	Carcinoma	0	0	N/A
A9	Breast	Malignant primary	Carcinoma	0	0	N/A
B1	Brain	Malignant primary	Neurologic tumours	0	0	N/A
B2	Brain	Low-grade neoplasia with malignant potential	Neurologic tumours	0	0	N/A
B3	Brain	Low-grade neoplasia with malignant potential	Neurologic tumours	0	0	N/A
B4	Brain	Low-grade neoplasia with malignant potential	Neurologic tumours	0	0.5	N/A
B5	Cervix	Malignant primary	Carcinoma	0	0.5	N/A
B6	Cervix	Malignant primary	Carcinoma	0	0	N/A
B7	Colon	Malignant primary	Carcinoma	0	0	N/A
B8	Colon	Malignant primary	Carcinoma	0	0	N/A
B9	Oesophagus	Malignant primary	Carcinoma	0	0	N/A
B10	Oesophagus	Malignant primary	Carcinoma	0	0.25	N/A
C1	Head and neck	Malignant primary	Carcinoma	0	0	N/A
C2	Tonsil	Malignant primary	Carcinoma	0	0	N/A
C3	Kidney	Benign tumour	Other	0	0	N/A
C4	Kidney	Malignant primary	Carcinoma	0	0.5	N/A
C5	Liver	Malignant primary	Carcinoma	0	0.25	N/A

Core ID	Tissue type	Diagnosis	Cancer classification	Stain intensity	Background	Comments
C6	Liver	Malignant primary	Carcinoma	0	0	N/A
C7	Lymph node	Malignant primary	Lymphoma	0	0.25	N/A
C8	Lymph node	Malignant primary	Lymphoma	0	0.25	N/A
C9	Lymph node	Malignant primary	Lymphoma	0	0	N/A
D1	Lung	Malignant primary	Carcinoma	0	0	N/A
D2	Lung	Malignant primary	Carcinoma	0	0.25	N/A
D3	Lung	Malignant primary	Carcinoma	0	0	N/A
D4	Gastric	Malignant primary	N/A	0	0	N/A
D5	Pleura	Low-grade neoplasia with malignant potential	Other	0	0.25	N/A
D6	Muscle	Malignant primary	Sarcoma	N/A	N/A	No core present
D7	Skeletal muscle	Benign tumour	N/A	0	0	N/A
D8	Ovary	Low-grade neoplasia with malignant potential	Other	0	0.25	
D9	Ovary	Malignant primary	Carcinoma	0	0.25	
D10	Ovary	Benign tumour	N/A	0	0.25	
E1	Pancreas	Malignant primary	N/A	0	0	
E2	Pancreas	Malignant primary	Carcinoma	0	0	
E3	Abdomen	Malignant primary	N/A	0	0	N/A
E4	Nerve	Low-grade neoplasia with malignant potential	Other	0.5	0	N/A
E5	Prostate	Malignant primary	Carcinoma	3	0	N/A
E6	Prostate	Malignant primary	Carcinoma	3	0	N/A
E7	Salivary gland	Benign tumour	N/A	0	0.25	N/A
E8	Salivary gland	Benign tumour	N/A	0	0.25	N/A
E9	Small intestine	Malignant primary	Carcinoma	0	0	N/A
E10	Small intestine	Malignant primary	Sarcoma	0	0	N/A
F1	Skin	Malignant primary	Sarcoma	0	0	N/A
F2	Soft tissue	Malignant primary	Sarcoma	0.75	0	N/A

Core ID	Tissue type	Diagnosis	Cancer classification	Stain intensity	Background	Comments
F3	Spleen	Malignant primary	Lymphoma	0	0	N/A
F4	Stomach	Malignant primary	Carcinoma	0	0.25	N/A
F5	Stomach	Malignant primary	Sarcoma	0	0	N/A
F6	Testis	Malignant primary	Carcinoma	0.25	0	N/A
F7	Testis	Malignant primary	Carcinoma	0.25	0	N/A
F8	Thyroid	Malignant primary	Carcinoma	0	0.25	N/A
F9	Thyroid	Malignant primary	Carcinoma	0	0.25	N/A
G1	Uterus	Malignant primary	Carcinoma	0	0	N/A
G2	Uterus	Malignant primary	Carcinoma	0	0	N/A
G3	Uterus	Benign tumour	N/A	0	0	N/A
G4	Soft tissue	Malignant primary	Sarcoma	0	0	N/A
G5	Skin	Malignant primary	Carcinoma	0	0	N/A
G6	Skin	Malignant primary	Carcinoma	0	0	N/A
G7	Skin	Malignant primary	Carcinoma	0	0	N/A
G10	Tonsil	Normal tissue	N/A	0	0	N/A

fuc-PSA, α-1,6-fucosylated PSA; IHC, immunohistochemical; mAb, monoclonal antibody; N/A, not available.

Supplementary Table 3. IHC staining analyses with anti-fuc-PSA mAb (2E9) on non-neoplastic tissue microarray.

Core ID	Tissue type	Stain intensity	Background	Comments
A1	Adrenal gland	0	0	N/A
A2	Adrenal gland	0	0.25	Background in stroma
A3	Adrenal gland	0	0	N/A
A4	Appendix	N/A	0.25	No epithelium present; background in luminal debris
A5	Appendix	0	0.25	Background in luminal debris
A6	Appendix	0	0.25	Background in luminal debris
A7	Bladder	0	0	No epithelium present
A8	Bladder	0	0.5	Background in luminal debris
A9	Bladder	N/A	N/A	No epithelium present;
B1	Bone marrow	0	0	N/A
B2	Bone marrow	0	0	N/A
B3	Bone marrow	0	0	N/A
B4	Breast	0	0.25	Background in luminal debris
B5	Breast	0	0.5	N/A
B6	Breast	0	0	N/A
B7	Cerebellum	0	0	N/A
B8	Cerebellum	0	0	N/A
B9	Cerebellum	0	0	N/A
C1	Cerebrum	0	0	N/A
C2	Cerebrum	0	0	N/A
C3	Cerebrum	0	0	N/A
C4	Cervix	0	0	N/A
C5	Cervix	0	0	N/A
C6	Cervix	0	0.25	Background in luminal debris
C7	Colon	0	0.25	Background in luminal debris and mucin
C8	Colon	0	0.25	Background in luminal debris and mucin

Core ID	Tissue type	Stain intensity	Background	Comments
C9	Colon	0	0.25	Background in luminal debris and mucin
D1	Oesophagus	0	0.25	Background in stroma
D2	Oesophagus	0	0	N/A
D3	Oesophagus	0	0.25	Background in luminal debris
D4	Heart	0	0	N/A
D5	Heart	0	0	N/A
D6	Heart	0	0	N/A
D7	Kidney	0.25	0.5	Weak staining in cortical tubules; background in vessel debris
D8	Kidney	0.25	0	N/A
D9	Kidney	0	0.5	Background in myoglobin casts; luminal debris and lining vessel
E1	Liver	0	0	N/A
E2	Liver	0.25	0	Faint staining in hepatocytes
E3	Liver	0.25	0.5	Faint staining in hepatocytes; background in luminal debris
E4	Lung	0	0.5	Background in luminal debris and macrophages
E5	Lung	0	0.25	Background in luminal debris
E6	Lung	0	0.5	Background in luminal debris
E7	Lymph node	0	0	N/A
E8	Lymph node	0	0.25	Background in luminal debris
E9	Lymph node	N/A	N/A	No lymphoid tissue on core
F1	Fallopian tube	0	0.5	Background in luminal debris
F2	Fallopian tube	0	0	N/A
F3	Fallopian tube	0	0.25	Background in luminal debris
F4	Ovary	0	0.5	Background in luminal debris
F5	Ovary	0	0	N/A

Core ID	Tissue type	Stain intensity	Background	Comments
F6	Ovary	0	0.25	Background in luminal debris
F7	Pancreas	0	0.25	Background in luminal debris
F8	Pancreas	0	0	N/A
F9	Pancreas	0.25	0.25	Faint staining in Islet cells; background in luminal debris
F10	Tonsil	0	0.25	Background in luminal debris

fuc-PSA, α-1,6-fucosylated PSA; IHC, immunohistochemical; mAb, monoclonal antibody; N/A, not available.
